# Induction of Daptomycin Tolerance in Enterococcus faecalis by Fatty Acid Combinations

**DOI:** 10.1128/AEM.01178-20

**Published:** 2020-10-01

**Authors:** William Brewer, Johnathan Harrison, Holly E. Saito, Elizabeth M. Fozo

**Affiliations:** a Department of Microbiology, University of Tennessee, Knoxville, Tennessee, USA; b Pellissippi State Community College, Natural and Behavioral Sciences Department, Knoxville, Tennessee, USA; University of Tokyo

**Keywords:** *Enterococcus faecalis*, daptomycin, fatty acid, membrane fluidity

## Abstract

Enterococcus faecalis is a Gram-positive bacterium that normally exists as an intestinal commensal in humans but is also a leading cause of nosocomial infections. Previous work noted that growth supplementation with serum induced tolerance to membrane-damaging agents, including the antibiotic daptomycin. Specific fatty acids found within serum could independently provide tolerance to daptomycin (protective fatty acids), yet some fatty acids found in serum did not and had negative effects on enterococcal physiology (nonprotective fatty acids). Here, we measured a wide array of physiological responses after supplementation with combinations of protective and nonprotective fatty acids to better understand how serum induces daptomycin tolerance. When cells were supplemented with either nonprotective fatty acid, palmitic acid, or stearic acid, there were marked defects in growth and morphology, but these defects were rescued upon supplementation with either protective fatty acid, oleic acid, or linoleic acid. Membrane fluidity decreased with growth in either palmitic or stearic acid alone but returned to basal levels when a protective fatty acid was supplied. Daptomycin tolerance could be induced if a protective fatty acid was provided with a nonprotective fatty acid, and some specific combinations protected as well as serum supplementation. While cell envelope charge has been associated with tolerance to daptomycin in other Gram-positive bacteria, we concluded that it does not correlate with the fatty acid-induced protection we observed. Based on these observations, we conclude that daptomycin tolerance by serum is driven by specific, protective fatty acids found within the fluid.

**IMPORTANCE** With an increasing prevalence of antibiotic resistance in the clinic, we strive to understand more about microbial defensive mechanisms. A nongenetic tolerance to the antibiotic daptomycin was discovered in Enterococcus faecalis that results in the increased survival of bacterial populations after treatment with the drug. This tolerance mechanism likely synergizes with antibiotic resistance in the clinic. Given that this tolerance phenotype is induced by incorporation of fatty acids present in the host, it can be assumed that infections by this organism require a higher dose of antibiotic for successful eradication. The mixture of fatty acids in human fluids is quite diverse, with little understanding between the interplay of fatty acid combinations and the tolerance phenotype we observe. It is crucial to understand the effects of fatty acid combinations on E. faecalis physiology if we are to suppress the tolerance physiology in the clinic.

## Abstract (precis)

With an increasing prevalence of antibiotic resistance in the clinic, we strive to understand more about microbial defensive mechanisms. A nongenetic tolerance to the antibiotic daptomycin was discovered in Enterococcus faecalis that results in the increased survival of bacterial populations after treatment with the drug. This tolerance mechanism likely synergizes with antibiotic resistance in the clinic. Given that this tolerance phenotype is induced by incorporation of fatty acids present in the host, it can be assumed that infections by this organism require a higher dose of antibiotic for successful eradication. The mixture of fatty acids in human fluids is quite diverse, with little understanding between the interplay of fatty acid combinations and the tolerance phenotype we observe. It is crucial to understand the effects of fatty acid combinations on E. faecalis physiology if we are to suppress the tolerance physiology in the clinic.

## INTRODUCTION

Enterococcus faecalis is a Gram-positive bacterium known primarily as a commensal of the mammalian intestine (1). In immunocompromised individuals, however, it can cause a variety of complications, including surgical wound and urinary infections, endocarditis, and bacteremia (2). E. faecalis is also resilient when exposed to a variety of stressors, allowing it to survive outside the body for extended periods of time, which likely increases transfer to patients in a hospital setting (1–4). Further, E. faecalis is resistant to a variety of antibiotics, complicating treatment strategies (4, 5).

To combat drug-resistant enterococcal infections, clinicians have utilized the antibiotic daptomycin (reviewed in references 6 to 8). This drug is thought to insert into phosphatidylglycerol-dominated portions of the cell membrane in a calcium-dependent manner, leading to ion leakage and cell death. Despite the success of daptomycin, clinical resistance has been reported (6, 9). Studies have also shown that the growth environment of E. faecalis can induce a physiological tolerance to this antibiotic (10–12). Specifically, growth of E. faecalis in the presence of bile and serum, mimicking its lifestyle as a commensal and pathogen, respectively, led to protection from daptomycin. This tolerance was induced by specific fatty acids found within bile or serum (11). This priming also protected E. faecalis from high concentrations of human bile and sodium dodecyl sulfate (SDS), demonstrating that protection was not specific to daptomycin. Further, this protection was not due to the selection of genetic mutants but, rather, to altered cellular physiology (11, 12).

Examination of the membrane content of E. faecalis after serum supplementation revealed a profile dominated by stearic acid (C_18:0_), linoleic acid (C_18:2_ *_cis_*_9,12_), oleic acid (C_18:1_ *_cis_*_9_), and palmitic acid (C_16:0_) (10, 11). The increase in these four species led to a decreased proportion of other native fatty acids in the membrane, most notably *cis*-vaccenic acid (C_18:1_ *_cis_*_11_). Additional analysis showed that only supplementation with oleic acid or linoleic acid provided protection from membrane stress agents even though stearic and palmitic acids are found natively in the membrane of E. faecalis (11, 12).

Not only did stearic acid and palmitic acid fail to induce daptomycin tolerance, but they also increased generation time significantly from times for control cultures, and the morphology of these cells was greatly perturbed (12). However, the failure to protect from daptomycin challenge was not due to a simple reduction in growth rate: the addition of linoleic acid alone could protect cultures from the antibiotic and also increased generation time (11, 12). Based on the differences in daptomycin tolerance observed when a fatty acid is independently provided to cultures, we chose to define any exogenously supplied fatty acid that induces daptomycin tolerance a protective fatty acid and all other fatty acids as nonprotective fatty acids.

It is interesting that human serum is effective at conferring daptomycin tolerance despite containing the nonprotective fatty acids stearic and palmitic acids. We hypothesized that the eukaryotic fatty acids oleic acid and linoleic acid drive a membrane-protective response even in the presence of nonprotective fatty acids. Within this work, we show that the supplementation of protective and nonprotective fatty acids in combination induces daptomycin tolerance in E. faecalis without observable negative physiological effects.

## RESULTS

### Protective fatty acids rescue E. faecalis from the negative growth effects of nonprotective fatty acids.

When grown in human serum, E. faecalis OG1RF had generation times similar to those of control cultures, despite containing palmitic and stearic acids that, if added independently, significantly impaired growth (10–12). To determine whether the presence of the protective fatty acids found in serum, namely, oleic and linoleic acids, could ameliorate the negative growth impacts of palmitic and stearic acids, we simulated the fatty acid membrane profile upon serum supplementation by adding each of these fatty acids to the culture (referred to as SLOP, i.e., stearic acid, linoleic acid, oleic acid, and palmitic acid). Note that as the concentration of each specific fatty acid varies in serum given the individual and the testing method, we opted to use 5 μg ml^−1^ as this concentration is below what has been measured or used in other *in vitro* studies (13–16).

**FIG 1:**
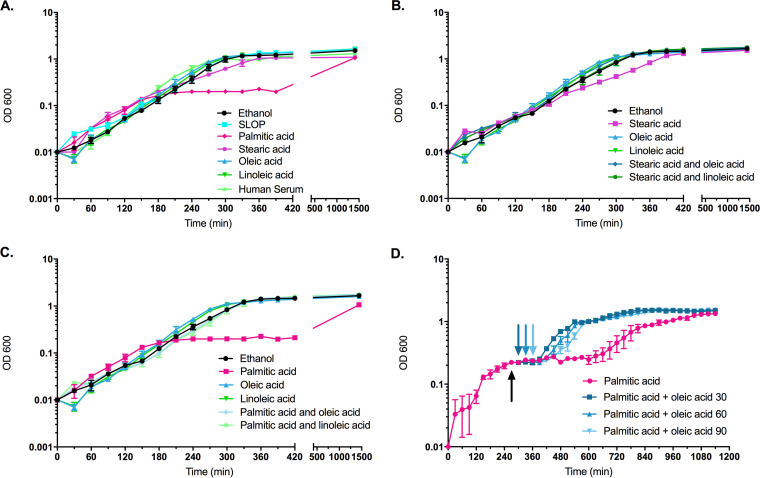
Addition of a protective fatty acid rescues growth defects associated with palmitic acid or stearic acid. All fatty acids were added to a final concentration of 5 μg ml^−1^, and ethanol (solvent control) was added to an equivalent final volume. (A) Addition of SLOP (stearic acid, linoleic acid, oleic acid, and palmitic acid), human serum (15%), or individual fatty acids as indicated. (B) Addition of stearic acid combinations. (C) Addition of palmitic acid combinations. (D) Palmitic acid was added at the time of dilution. Stasis is indicated by the black arrow. Oleic acid was added (indicated by arrows) at 30, 60, and 90 min poststasis (indicated according to the color legend). Note, data from the same biological replicates are replotted in panels B and C. For all experiments, *n* = 3.

The addition of SLOP to OG1RF cultures had no impact on generation time compared to that of the solvent control or serum supplementation of cultures (Fig. 1A and Table 1; see also Table S1 in the supplemental material), unlike the addition of either palmitic or stearic acid alone (see below). To confirm that the fatty acids were taken up by OG1RF, we analyzed the membrane fatty acid content via gas chromatography of fatty acid methyl esters (GC-FAME) (see Materials and Methods). The membrane content of SLOP cultures had an increased proportion of palmitic acid (C_16:0_) over that of the solvent control, and also contained oleic acid (C_18:1_ *_cis_*_9_) and linoleic acid (C_18:2_ *_cis_*_9,12_). As expected, there was a significant reduction in the natively produced *cis*-vaccenic acid (C_18:1_ *_cis_*_11_); however, the amount of stearic acid (C_18:0_) trended lower in SLOP cultures than in the solvent control (Table 2 and Table S2) (see Discussion).

**TABLE 1:** Generation times of OG1RF grown in BHI broth and the indicated supplement(s)

Medium constituent(s)^*a*^	Generation time (min)^*b*^
Solvent control (ethanol)	41.85 ± 0.91
Single fatty acids	
C_18:1_ *_cis_*_9_	34.91 ± 1.03
C_18:2_ *_cis_*_9,12_	37.07 ± 0.97
C_16:0_	NA
C_18:0_	64.79 ± 5.77
Fatty acid combinations	
C_18:1_ *_cis_*_9_ + C_16:0_	43.35 ± 0.67
C_18:1_ *_cis_*_9_ + C_18:0_	43.61 ± 0.85
C_18:2_ *_cis_*_9,12_ + C_16:0_	40.17 ± 0.51
C_18:2_ *_cis_*_9,12_ + C_18:0_	39.34 ± 0.84
SLOP	37.87 ± 1.05
Human serum	41.32 ± 0.97

a C_18:1_ *_cis_*_9_, oleic acid; C_18:2_ *_cis_*_9,12_, linoleic acid; C_16:0_, palmitic acid; C_18:0_, stearic acid; SLOP, stearic, linoleic, oleic, and palmitic acids. Ethanol (solvent control) was added to a final concentration of 0.2%. Each fatty acid, alone or in combination, was supplemented to a final concentration of 5 μg ml^−1^. Human serum was supplemented to a final concentration of 15%.

b Values are averages ± standard deviations for *n *= 3 biological replicates (*n *= 6 for stearic acid due to variability). Statistical analyses are found in Table S1 in the supplemental material. NA, not available.

**TABLE 2:** OG1RF membrane composition after long-term fatty acid supplementation

Fatty acid(s)	Membrane composition (%) after supplementation with:^*a*^									
	Ethanol	C_18:1_ *_cis_*_9_	C_18:2_ *_cis_*_9,12_	C_16:0_	C_18:0_	C_18:1_ *_cis_*_9_ + C_16:0_	C_18:1_ *_cis_*_9_ + C_18:0_	C_18:2_ *_cis_*_9,12_ + C_16:0_	C_18:2_ *_cis_*_9,12_ + C_18:0_	SLOP
C_14:0_	4.59 ± 0.014	2.23 ± 0.058	2.53 ± 1.03	1.99 ± 0.07	3.94 ± 0.21	0.63 ± 0.07	3.23 ± 0.12	0.56 ± 0.04	2.62 ± 0.59	0.68 ± 0.07
C_16:1_ *_cis_*_9_	5.99 ± 0.021	3.08 ± 0.057	3.44 ± 0.26	3.27 ± 0.12	4.69 ± 0.13	1.12 ± 0.11	3.63 ± 0.17	0.87 ± 0.08	2.30 ± 0.42	ND
C_16:0_	42.59 ± 0.36	10.18 ± 3.78	9.81 ± 4.60	81.45 ± 0.94	22.27 ± 2.56	59.87 ± 0.27	13.36 ± 2.33	56.11 ± 0.51	6.91 ± 3.25	50.13 ± 2.58
C_18:2_	ND	ND	73.68 ± 10.29	ND	0.19 ± 0.04	ND	ND	38.45 ± 0.15	49.14 ± 4.52	7.14 ± 1.66
C_18:1_ *_cis_*_9_	0.66 ± 0.010	71.81 ± 8.18	1.70 ± 0.49	0.97 ± 0.2	0.42 ± 0.08	32.24 ± 0.94	57.31 ± 5.05	0.87 ± 0.03	0.80 ± 0.07	34.46 ± 0.85
C_18:1_ *_cis_*_11_	36.15 ± 0.061	5.16 ± 2.32	4.80 ± 2.63	9.09 ± 0.58	19.54 ± 2.38	0.95 ± 0.05	7.11 ± 1.83	0.94 ± 0.04	3.69 ± 1.89	1.24 ± 0.10
C_18:0_	6.42 ± 0.026	1.84 ± 0.07	1.79 ± 0.16	3.22 ± 0.19	46.92 ± 4.48	1.39 ± 0.02	8.13 ± 0.19	1.00 ± 0.09	31.63 ± 2.60	2.56 ± 0.24
Other^*b*^	3.60 ± 0.08	5.52 ± 0.72	1.71 ± 1.68	ND	2.09 ± 0.08	3.72 ± 0.89	7.09 ± 0.54	0.24 ± 0.34	1.86 ± 0.45	3.55 ± 0.70
SFA/UFA^*c*^	1.21 ± 0.02	0.20 ± 0.06	0.19 ± 0.09	6.52 ± 0.40	2.82 ± 0.30	1.65 ± 0.03	0.37 ± 0.05	1.40 ± 0.02	0.76 ± 0.06	1.15 ± 0.13

a C_18:1_ *_cis_*_9_, oleic acid; C_18:2_ *_cis_*_9,12_, linoleic acid; C_16:0_, palmitic acid; C_18:0_, stearic acid; SLOP, stearic, linoleic, oleic, and palmitic acids. Ethanol (solvent control) was added to a final concentration of 0.2%. Each fatty acid, alone or in combination, was supplemented to a final concentration of 5 μg ml^−1^. Percentages of total membrane content were determined by GC-FAME by Microbial ID, Inc. Data are the averages ± standard deviations from three independent experiments. ND, not determined. Statistical analyses are found in Table S2 in the supplemental material.

b Total of all fatty acids comprising <3% of the total membrane content.

c SFA/UFA, ratio of total saturated fatty acids to total unsaturated fatty acids.

We then compared growth of OG1RF with either a single nonprotective fatty acid or paired with a protective fatty acid. When stearic acid was added independently to cultures, the generation time was increased compared to that of the control cultures (Table 1) (*P* < 0.0001). This was probably because when added alone, stearic acid was over 46% of the total membrane composition, increasing the saturated/unsaturated fatty acid ratio and rigidifying the membrane (Table 2) (see below). However, when cultures were supplemented with both stearic and oleic acids, not only was growth restored to that of control cultures but also there was notably far less stearic acid found in the membrane (approximately 8%; *P < *0.0001) than if only stearic acid was provided; indeed, this proportion of the membrane was not significantly different from that of solvent control cultures (Table 2 and Table S2; see also Discussion). When provided both stearic and linoleic acids, OG1RF had a generation time to similar to that of control cultures (Table 1 and Table S1). In this case, however, stearic acid comprised approximately 31% of the total content, far higher than the proportion in the control (*P < *0.0001) or when cultures were supplemented with both stearic acid and oleic acid (*P < *0.0001) (Table 2 and Table S2; see also Discussion).

When only palmitic acid was given to cells, OG1RF entered an early stasis period (Fig. 1A and C) and reached stationary phase only after selection of a suppressor mutant population (12; data not shown) (see Discussion); yet palmitic acid was a major constituent of the membrane profile in control cultures (Table 2). When palmitic acid was provided exogenously, over 81% of the membrane was comprised of this saturated fatty acid, resulting in a high saturated/unsaturated fatty acid ratio (Table 2). When palmitic acid was supplemented together with oleic acid, the percentage of palmitic acid in these cultures was much less than if it was given alone but was still significantly higher than that of control cultures (approximately 60% versus 43%; *P < *0.0001) (Table 2 and Table S2). Similar trends were seen when linoleic acid was provided with palmitic acid to cultures: cellular growth resembled that of control cultures or of those supplemented with linoleic acid alone (Table 1, Fig. 1C, and Table S1). Examination of the membrane profile also showed decreased amounts of palmitic acid if linoleic acid was provided simultaneously to OG1RF compared to levels in cultures given palmitic acid alone, but levels were still higher than those of control cultures (*P* < 0.001) (Table 2; Table S2).

Given that the addition of oleic acid could ameliorate the negative consequences of palmitic acid on growth, we tested whether oleic acid could restore growth to cultures that entered a palmitic acid-induced stasis. We supplied palmitic acid after cultures were diluted, and once stasis was established, oleic acid was added. By supplementing oleic acid as late as 90 min poststasis, the culture was able to resume growth (Fig. 1D). Redilution of these cells indicated that oleic acid did not select for a genetic mutant isolate as they were unable to grow in the presence of palmitic acid when challenged again (data not shown). Given the vast array of growth impacts and the altered levels of fatty acid incorporation within the membrane, we next examined the morphological consequences of these supplements on OG1RF.

### Protective fatty acids prevent negative morphological effects from saturated fatty acids.

Growth in serum has little impact on the E. faecalis growth rate (10, 12; see above) or cellular morphology. However, individual supplementation with either stearic acid or palmitic acid caused severe distortion of cellular morphology (11, 12). We hypothesized that serum has little impact on morphology because oleic acid and linoleic acid are able to prevent severe cellular distortion caused by stearic or palmitic acid.

**FIG 2:**
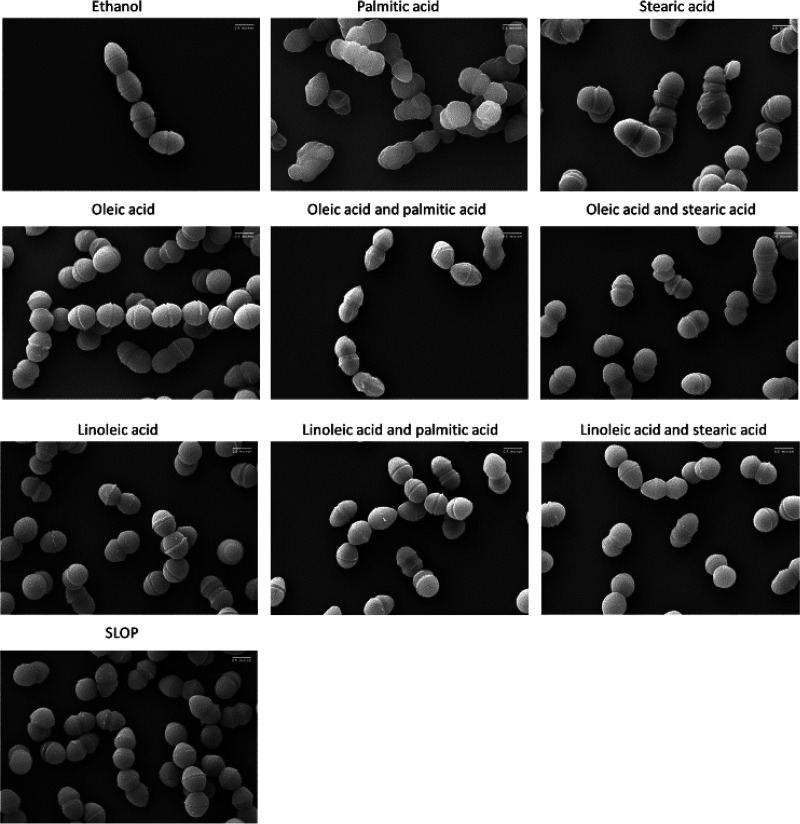
Fatty acids induce altered morphology of OG1RF. Scanning electron micrographs of exponential-phase cells after long-term supplementation with fatty acids (5 μg ml^−1^ of each) or solvent control (ethanol) are shown. Images were taken at a magnification of 45,000 and at 5.0 keV. Scale bar, 0.5 μm. Sample images of *n *= 2 biological replicates are shown, and a minimum of 10 fields per replicate were observed.

OG1RF cultures supplemented with SLOP displayed a relatively typical diplococcal morphology compared to that of controls (Fig. 2; Fig. S1). We did note that there was an overall reduction in cellular length (Fig. S2) compared to that of control cultures. However, cellular morphology was highly consistent across the culture (Fig. S1).

When given independently, the protective fatty acids oleic acid and linoleic acid also led to a reduction in overall cellular length (Fig. S2), but otherwise the morphology resembled that of control cultures. Cells supplemented with palmitic acid were severely distorted in their shape, oftentimes with little or no distinction from one cell to the next. Unlike the characteristic diplococcus shape, we observed what appeared to be curved cells branching from nonpolar ends, reminiscent of expanding dough (Fig. 2). Cells supplemented with palmitic acid in conjunction with oleic acid or linoleic acid, however, had a morphology similar to that of control cells, with apparent normal septum placement, though they were reduced in overall cell length (Fig. S2).

Stearic acid supplementation also led to apparent misplacement of septa and improper cellular division, similar to characteristics of palmitic acid-supplemented cells (Fig. 2). The cells also appeared wrinkled, which may be due to cell wall defects (see Discussion). However, when stearic acid was given in combination with oleic or linoleic acids, cellular morphology was similar to that of control cells. As seen with other fatty acid combinations, the addition of either protective fatty acid led to an overall reduction of cellular length (Fig. S2). In combinations, oleic or linoleic acid prevented the negative cellular morphology associated with supplementation of the saturated, nonprotective fatty acids palmitic and stearic acid.

### Addition of protective fatty acids allow for growth in the presence of a *de novo* fatty acid biosynthesis inhibitor.

Previous work has demonstrated that E. faecalis can overcome inhibition of *de novo* fatty acid biosynthesis if grown in the presence of human serum, oleic acid, or linoleic acid but not of palmitic or stearic acid (12, 17). We thus examined if fatty acid combinations could support growth of E. faecalis in the presence of the *de novo* fatty acid biosynthesis inhibitor cerulenin.

As shown, cells grown in the presence of cerulenin and our synthetic serum fatty acid mixture, SLOP, grew as well as cultures lacking the drug (Fig. S3). Further, as long as either oleic or linoleic acid was present in cultures, OG1RF supplemented with a nonprotective fatty acid was also able to grow in the presence of cerulenin (Fig. S3). We therefore conclude that unsaturated fatty acids are required for proper membrane function and cellular viability.

### Fatty acid supplementation causes a varied response to cell envelope charge.

An increase in overall cellular charge via elevated levels of the positively charged phospholipid lysl-phosphatidylglycerol has been associated with a reduction in ion leakage caused by cationic antimicrobial peptides in Staphylococcus aureus (18–20) and increased resistance to daptomycin in Bacillus subtilis (21). We observed alterations in the polar head group composition of OG1RF dependent upon supplementation with oleic or linoleic acid (data not shown), and the addition of either could induce daptomycin tolerance (11, 12). Thus, we examined whether addition of exogenous fatty acids resulted in an altered overall cellular charge via interactions with the positively charged protein cytochrome *c* (22).

Surprisingly, not all conditions impacted cellular charge in comparison to results in control cultures (Fig. S4). The addition of SLOP caused a modest but significant increase in cell charge (*P < *0.0001), as indicated by the larger amount of unbound cytochrome *c*. Supplementation with oleic acid caused no change relative to the level in the control, while growth in linoleic acid caused a significant decrease in cell charge (*P < *0.05). Examination of the nonprotective fatty acids also did not correlate with cellular charge differences: stearic acid supplementation alone resembled that of control cultures, whereas the addition of palmitic acid led to a significant increase in charge (*P < *0.05).

### Nonprotective fatty acids alter membrane fluidity.

The cellular membrane adjusts its fluidity upon changing environmental conditions to maintain both a protective barrier and functioning membrane proteins (23, 24). Alterations, then, in the ratio of saturated to unsaturated fatty acids may impact membrane fluidity and the subsequent effectiveness of daptomycin. We performed anisotropy using the dye DPH (1,6-diphenyl-1,3,5-hexatriene) to determine membrane fluidity on protoplasts to ensure proper interaction of the dye with the cellular membrane (25). Further, given the severe growth effects for some fatty acid supplements, we performed short-term supplementation in which exponential cultures were exposed to fatty acids for 30 min (12; also Materials and Methods). As noted previously, short-term supplementation led to altered membrane composition from that of the control (Table 3 and Table S2); overall, however, exogenous fatty acids did not comprise as large a portion of total membrane content as they did when cells were supplemented with fatty acids from dilution, similar to what was noted previously with long-term supplementation (Table 2) (12).

**TABLE 3:** OG1RF membrane composition after short-term fatty acid supplementation

Fatty acid(s)	Membrane composition (%) after supplementation with:^*a*^									
	Ethanol	C_18:1_ *_cis_*_9_	C_18:2_ *_cis_*_9,12_	C_16:0_	C_18:0_	C_18:1_ *_cis_*_9_ + C_16:0_	C_18:1_ *_cis_*_9_ + C_18:0_	C_18:2_ *_cis_*_9,12_ + C_16:0_	C_18:2_ *_cis_*_9,12_ + C_18:0_	SLOP
C_14:0_	3.61 ± 0.61	4.51 ± 0.35	2.98 ± 0.59	2.06 ± 0.29	3.86 ± 0.32	3.03 ± 0.44	4.44 ± 0.52	3.20 ± 0.14	3.61 ± 0.42	3.05 ± 0.03
C_16:1_ *_cis_*_9_	5.04 ± 0.60	4.26 ± 0.36	4.00 ± 0.59	2.88 ± 0.28	4.47 ± 0.36	3.61 ± 0.51	4.56 ± 0.42	4.43 ± 0.23	4.66 ± 0.28	3.60 ± 0.19
C_16:0_	42.09 ± 0.13	28.48 ± 1.60	35.50 ± 2.07	65.20 ± 1.84	28.40 ± 0.82	42.74 ± 0.98	24.33 ± 0.032	44.97 ± 1.22	28.85 ± 0.66	46.88 ± 0.68
C_18:2_	ND	ND	19.88 ± 5.80	ND	ND	ND	ND	13.87 ± 1.54	25.96 ± 1.10	3.97 ± 0.90
C_18:1_ *_cis_*_9_	0.55 ± 0.05	34.01 ± 3.37	0.78 ± 0.10	0.48 ± 0.04	0.41 ± 0.03	27.02 ± 2.24	34.87 ± 3.83	0.85 ± 0.07	0.80 ± 0.06	8.42 ± 0.68
C_18:1_ *_cis_*_11_	37.21 ± 0.19	20.21 ± 0.99	26.96 ± 2.01	20.70 ± 1.28	24.92 ± 0.86	17.84 ± 1.74	19.73 ± 0.27	25.27 ± 0.62	24.86 ± 0.11	21.40 ± 1.12
C_18:0_	7.47 ± 1.49	3.98 ± 0.72	6.10 ± 0.40	6.44 ± 1.31	35.50 ± 2.15	3.54 ± 0.05	8.88 ± 2.29	4.85 ± 0.24	8.09 ± 0.60	6.76 ± 0.05
Other^*b*^	3.93 ± 0.09	3.44 ± 1.84	3.79 ± 0.88	2.13 ± 0.17	2.44 ± 0.30	2.12 ± 0.28	3.13 ± 0.53	2.56 ± 0.46	3.16 ± 0.60	5.80 ± 0.89
SFA/UFA^*c*^	1.17 ± 0.04	0.61 ± 0.06	0.83 ± 0.09	2.90 ± 0.14	2.16 ± 0.13	1.00 ± 0.03	0.63 ± 0.08	1.17 ± 0.06	0.70 ± 0.01	1.37 ± 0.04

a C_18:1_ *_cis_*_9_, oleic acid; C_18:2_ *_cis_*_9,12_, linoleic acid; C_16:0_, palmitic acid; C_18:0_, stearic acid; SLOP, stearic, linoleic, oleic, and palmitic acids. Ethanol (solvent control) was added to a final concentration of 0.2%. Each fatty acid, alone or in combination, was supplemented to a final concentration of 5 μg ml^−1^. Percentages of total membrane content were determined by GC-FAME by Microbial ID, Inc. Data are the averages ± standard deviations from three independent experiments. ND, not determined. Statistical analyses are found in Table S2 in the supplemental material.

b Total of all fatty acids comprising <3% of the total membrane content.

c SFA/UFA, ratio of total saturated fatty acids to total unsaturated fatty acids.

**FIG 3:**
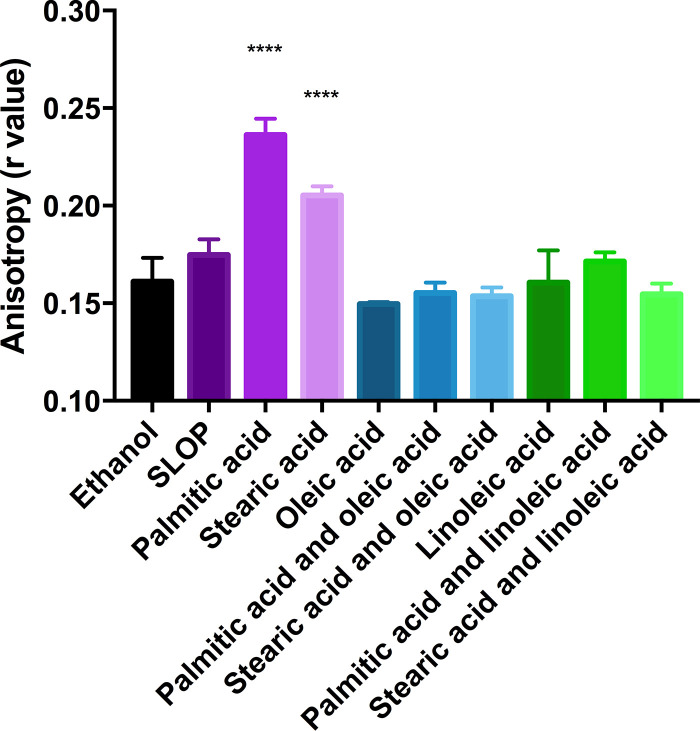
Membrane fluidity of OG1RF after short-term supplementation with fatty acids. Cells were grown to mid-log phase supplemented either with 5 μg ml^−1^ of each fatty acid, 15% human serum, or an equivalent volume of ethanol (solvent control) for 30 min. Upon formation of protoplasts, anisotropy was determined using DPH at an excitation wavelength of 350 nm and emission wavelength of 428 nm. ****, *P* < 0.0001, as determined via Tukey’s range test (*n* = 3).

The fluidity of cultures supplemented with SLOP resembled that of the solvent control (Fig. 3). Further, cells exposed to oleic acid or linoleic acid had fluidity similar to that of each other and control cultures. Cells supplemented solely with either of the saturated fatty acids palmitic or stearic acid gave a statistically significant higher *r* value (*P < *0.0001), indicative of a more rigid membrane than that of the control and all other conditions examined (Fig. 3). This correlated well with the saturated/unsaturated fatty acid ratio of such cells (Table 3). Combining a protective and nonprotective fatty acid, we noted that membrane fluidity returned to that of control cultures (Fig. 3). Thus, while saturated fatty acids rigidified the overall membrane, growth with protective fatty acids appeared to have no impact (see Discussion).

### Protection from daptomycin by beneficial fatty acid supplements is not hindered by the presence of palmitic or stearic acids.

Our data are supportive of protective fatty acids driving cellular physiology when they are given with nonprotective fatty acids. Therefore, we examined the induction of daptomycin tolerance by our mixtures (12).

**FIG 4:**
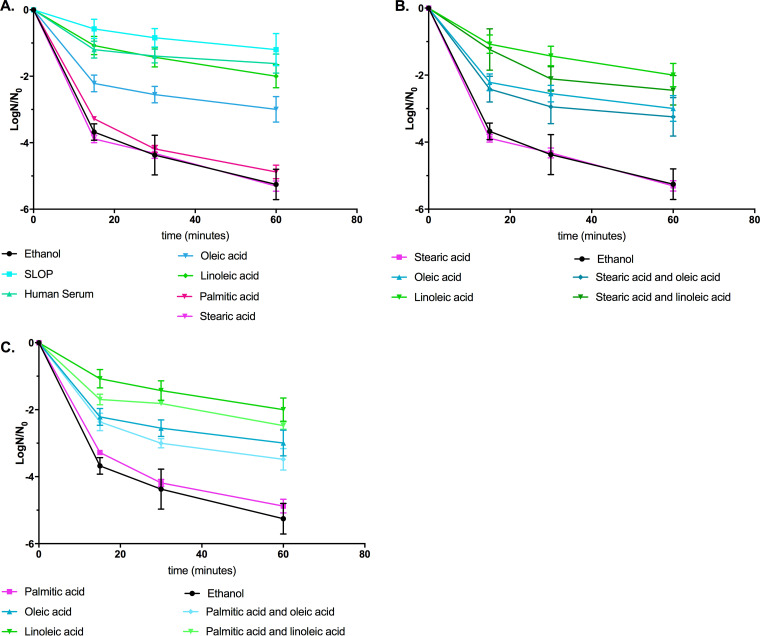
Oleic or linoleic acid can induce daptomycin tolerance in the presence of nonprotective fatty acids. (A) Supplementation with SLOP (stearic acid, linoleic acid, oleic acid, and palmitic acid), human serum (15%), oleic acid, or linoleic acid led to an increase in the survival rate for all time points (*P < *0.0001). Cultures supplemented with oleic acid did not differ from cultures supplemented with oleic acid mixtures. (B) Supplementation with oleic acid or linoleic acid in combination with stearic acid led to an increase in the survival rate at all time points (*P < *0.0001). (C) Supplementation with oleic acid or linoleic acid in combination with palmitic acid led to an increase in the survival rate at all time points (*P < *0.0001). Note, data for the same biological replicates are replotted in the different panels (*n *= 3 biological replicates).

When comparing cells pregrown in either serum or SLOP, we saw a significant increase (*P < *0.0001) in fold protection over that of the controls (Fig. 4A). There was no statistical difference in the log ratio of survivors of serum versus that of SLOP-grown cells. Thus, protection against daptomycin via serum supplementation was likely due to the direct or indirect presence of protective fatty acids found within the mixture.

The addition of either oleic acid or linoleic acid led to a 2-log increase in survival after daptomycin treatment compared to survival in the solvent control (*P < *0.0001) (Fig. 4A). This was in contrast to supplementation with either palmitic or stearic acid, which had a survival rate similar to that of the solvent control (Fig. 4A). However, addition of oleic acid with either palmitic or stearic acid resulted in protection similar to that of oleic acid alone (Fig. 4B and C). Despite this, protection induced by human serum was significantly greater than that from the addition of either oleic acid or the oleic acid combinations (*P < *0.0001).

Supplementation with linoleic acid or a linoleic acid combination also led to a significant increase in the survival rate over that of the controls (*P < *0.0001). However, the addition of SLOP induced significantly higher tolerance than all supplements with the exception of human serum (*P < *0.05) (Fig. 4A). These findings reinforce the conclusion that host-derived fatty acids are sufficient to provide protection from daptomycin (10–12). These data also demonstrate that a single protective fatty acid is both necessary and sufficient for increased survival when cells are challenged with daptomycin in the presence of a nonprotective fatty acid.

## DISCUSSION

Previously, we had noted that growth of E. faecalis in either serum or bile resulted in increased proportions of palmitic, stearic, oleic, and linoleic acids within the membrane and induced tolerance to daptomycin (11). However, supplementation with individual fatty acids resulted in two distinctive phenotypes: daptomycin protective and nonprotective (11, 12). We found that we could simulate the effects of serum by supplementation with a mixture of the four dominant fatty acids (SLOP) found within the fluid. Further, a combination containing at least one protective fatty acid induced protection from daptomycin and prevented negative physiological effects that were observed after supplementation with a saturated, nonprotective fatty acid (Fig. 4). In particular, we noted that membrane fluidity was restored to control culture levels (Fig. 3), which likely improved the overall health of these cells. Changes in cellular charge did not correlate with restored cellular health and daptomycin tolerance, indicating that this feature may not be the main driver in E. faecalis for fatty acid-induced tolerance (see Fig. S3 in the supplemental material).

Supplementation of cells with either palmitic acid or stearic acid alone resulted in severe morphological deformities (Fig. 2). Cells appeared with altered placement of septa and clear problems with proper cellular division. This morphology is reminiscent of that of E. faecalis with nonfunctioning DivIVA, which localizes to cell poles and septa to mediate proper cell division (26). It is possible that the increased saturated fatty acids within the membrane led to DivIVA mislocalization (Fig. 1 and Table 1). This aberrant cell morphology was rescued by addition of either protective fatty acid, suggesting that unsaturated fatty acids are needed for proper membrane protein localization and/or function.

Cells that were supplemented with palmitic acid underwent initial rounds of cellular division (Fig. 1A and C) before growth stasis occurred. Why were the effects of palmitic acid delayed? Upon dilution back from stationary phase, cells contain their preformed lipid molecules with the optimal fatty acid tail composition. As cells grow, these preformed lipids will comprise a portion of the membrane while newly synthesized lipids are generated to ensure proper size of the daughter cell. E. faecalis will utilize the exogenously supplied palmitic acid to generate these new lipids, and eventually, the old lipids will be diluted out such that daughter cells contain new lipids harboring mainly palmitic acid tails (Table 2). This will lead to decreased membrane fluidity (Fig. 3) and growth stasis. Genetic suppressors can be isolated that escape this cellular stasis (11, 12). Experiments are ongoing to elucidate the mechanism(s) of suppression; it is likely that these isolates are defective in their use of exogenously supplied fatty acids or in their ability to repress *de novo* fatty acid biosynthesis (see below).

A recent investigation into the mechanism of daptomycin killing revealed that membrane fluid microdomains of Bacillus subtilis were disrupted, leading to delocalization of the membrane-associated machinery vital for cell division (27). Daptomycin is thought to decrease fluidity after insertion, so a more fluid membrane is theoretically less sensitive to the membrane-disrupting effects of this proposed mechanism (7). While we did not observe increased fluidity after protective fatty acid supplementation, it is possible that we encountered a technical lower limit while using anisotropy. Further, anisotropy measures total fluidity, not that of localized regions. While it is surprising that the addition of unsaturated fatty acids, which can alter the saturated/unsaturated fatty acid ratio, did not alter fluidity, perhaps alterations in membrane protein concentration and/or composition compensated for these changes, maintaining fluidity at normal levels.

Our fluidity data do provide some interesting insights into the basic membrane physiology of E. faecalis. Even a brief (30-min) exposure to saturated fatty acids led to decreases in membrane fluidity (Fig. 3). This suggests that E. faecalis does not have a system expressed under these conditions with which to increase membrane fluidity. Under a different physiological condition, B. subtilis can increase its membrane fluidity in response to cold shock through production of Des, a desaturase (23, 28). Des targets preformed lipids, forming unsaturated fatty acid bonds in saturated fatty acids. Its production is induced via the two-component system DesKR (23). DesK, a membrane-bound sensor kinase, has a conformation in the membrane that is sensitive to membrane compression upon decreased fluidity, stimulating a phosphorelay (29, 30). Again, while our cells did not experience cold shock, the rigidification of their membranes through the addition of excess saturated fatty acids did not induce a similar pathway (Tables 2 and 3; Fig. 3). Note that Des is not annotated in the OG1RF genome, and our attempts to identify a Des homolog using PSI-BLAST as well as domain predictions were unsuccessful, suggesting that if E. faecalis does have a membrane-sensing protein, it is unique. Past analysis showed that OG1RF decreased its saturated/unsaturated fatty acid ratio in response to growth at low temperatures, but this likely occurred at the *de novo* biosynthesis level (11). Interestingly, increasing the proportion of unsaturated fatty acids within the membrane did not cause significant issues for growth and viability, suggesting that E. faecalis can adjust to a too fluid membrane though how it adjusts is not known (11, 12).

Other membrane alterations, besides fluidity, have implicated a role for the cell envelope in daptomycin repulsion. Increases in the cellular envelope charge appear to prevent drug interaction with the membrane and/or block the formation of pores (reviewed in references 6 to 8). Our own observations (data not shown) demonstrate that growth with oleic acid can lead to an increase in specific lysyl-phosphatidylglycerol species in the membrane, which could reduce the negative charge of the enterococcal cellular envelope. Enterococcal mutant strains lacking *mprF2* have greatly reduced lysyl-phosphatidylglycerol in the membrane and are more susceptible to cationic antimicrobial peptides (CAMPs) (31). Similarly, in S. aureus, upregulation of the *dlt* operon, which adds d-alanyl to lipoteichoic acid resulting in an increased cell wall charge, has been associated with tolerance to daptomycin (22, 32). However, under our conditions, there did not seem to be a correlation in overall cell charge and protection from daptomycin.

An unexpected finding of this study was the two different mechanisms by which oleic acid negates the growth effects associated with saturated fatty acid supplementation. When provided alone, stearic acid comprised a significantly larger portion of membrane composition than that in control cultures (Table 2). However, when stearic acid (C_18:0_) was given with oleic acid from lag phase, the amount found within the membrane was significantly lower than that in control cultures (Table 2). This implies that oleic acid blocks incorporation of stearic acid by OG1RF. Yet this does not seem to be the case for palmitic acid: when C_16:0_ was given with oleic acid, levels were still elevated above the level in the control (Table 2 and Table S2). Further, in SLOP-supplemented cells, the level of stearic acid was again found to be lower than that in the controls. When linoleic acid and stearic acid were provided together in the medium, the membrane composition for OG1RF showed a significant increase in stearic acid in the membrane (Table 2), supporting the idea that there is competition between stearic acid and oleic acid for use in the membrane.

How could oleic acid block stearic acid uptake in the membrane? In S. aureus and Streptococcus pneumoniae, exogenous fatty acids that associate with the membrane are bound by a specific FakB binding protein that, in concert with FakA, phosphorylates the fatty acid, activating it for use in lipid biosynthesis (33). Biochemical analyses suggest that there is a specific FakB binding protein for saturated fatty acids, another one for unsaturated fatty acids, and, in the case of S. pneumoniae, a third for polyunsaturated fatty acids (13, 34). As oleic acid addition seems to prevent utilization of stearic acid by OG1RF, this may suggest that the specificity of the FakB proteins in E. faecalis does not follow that of S. aureus or S. pneumoniae. Oleic acid has also been shown to repress *de novo* fatty acid biosynthesis in E. faecalis (35). Alternatively, then, the reduced amounts of stearic acid observed in cultures supplemented with SLOP or oleic and stearic acid may be due to transcriptional repression of *de novo* synthesis. Additional evidence supports a block in *de novo* synthesis: when cultures were supplemented for only 30 min with stearic and oleic acid, the levels of stearic acid were not nearly as reduced as during long-term supplementation (compare data in Tables 2 and 3).

Here, we showed that oleic acid and linoleic acid are able to rescue E. faecalis from the negative physiological effects of nonprotective fatty acids and are solely responsible for providing the protection observed after supplementation with human serum. The presence of either oleic or linoleic acid within fatty acid mixtures likely leads to improved membrane protein activities and functions that are sensitive to membrane fluidity (27, 36–41).

## MATERIALS AND METHODS

### Bacterial growth conditions.

E. faecalis OG1RF was grown statically at 37°C in brain heart infusion (BHI) broth for all experimental conditions. Overnight cultures were diluted to an optical density at 600 nm (OD_600_) of 0.01 before experimentation. For long-term supplementation, fatty acids were added at the time of dilution (11). For short-term supplementation, fatty acids were added to cultures during exponential phase, an OD_600_ of 0.25 (12). Unless otherwise noted, cells were harvested at an OD_600_ of 0.3 for long-term supplementation and 30 min after fatty acid addition for short-term supplementation. Growth was monitored by the OD_600_, and all fatty acids (Millipore-Sigma) were supplemented to a final concentration of 5 μg ml^−1^, as follows: oleic acid (C_18:1_ *_cis_*_9_) 17.7 μM; linoleic acid (C_18:2_ *_cis_*_9,12_) 17.82 μM; palmitic acid (C_16:0_) 19.49 μM; stearic acid (C_18:0_) 17.58 μM. Human serum (MP Biomedicals) was supplemented at a final concentration of 15%. Comparison of generation times was via Tukey’s range test (see Table S1 in the supplemental material).

### GC-FAME.

Cells were grown using both the long-term and short-term supplementation methods described above. Cultures (15 ml) were harvested by centrifugation at 2,739 × *g* for 10 min, washed extensively in 1× phosphate-buffered saline (PBS) twice, pelleted, and stored at −80°C. Gas chromatography of fatty acid methyl esters (GC-FAME) was performed by Microbial ID (Newark, DE) using methods described previously (42). Comparisons of fatty acid membrane profiles was via Tukey’s range test (Table S2).

### Scanning electron microscopy.

Bacterial cultures (50 ml) were grown using the long-term supplementation methods described above. The cells were harvested by centrifugation at 2,739 × *g* for 10 min, washed in 1× PBS, and fixed in 500 μl of 3% glutaraldehyde for 60 min. Cells were washed three times in 10 ml of sterile water, decanted, and resuspended in the remaining water. An aliquot (20 μl) of each sample was fixed to a 5- by 5-mm silicon chip with polylysine and then dehydrated using a series of ethanol washes (25, 50, 70, 95, and 100%) for 10 min each. After ethanol washes, the cells were placed in a Ladd critical point dryer for three cycles of 10 min each. The dried samples were then coated with iridium using a sputter coater and visualized using a Zeiss Auriga 40 instrument at the Center for Advanced Microscopy and Imaging at the University of Tennessee at 5.0 keV. Biological duplicates were performed for each growth condition, and a minimum of 10 fields were imaged for each sample. The average length of 30 cells (10 cells across 3 images) was determined via ImageJ, and statistical comparisons were made via Tukey’s range test.

### Determination of cellular charge.

The overall charge of the bacterial cells was determined using a previously described method with modifications (22). Bacterial cultures were grown using the short-term supplementation methods described above. Cells were washed twice with PBS, resuspended to a final OD_600_ of 3.0 in 3.0 ml of 20 mM 3-(*N*-morpholino) propanesulfonic acid (MOPS) with 1 mg ml^−1^ cytochrome *c* (Millipore-Sigma), and incubated at room temperature for 10 min. The samples were centrifuged at 2,739 × *g* for 10 min, the supernatant was extracted, and the OD_530_ was measured. The concentration of cytochrome *c* was determined via a standard curve of known cytochrome *c* concentrations. The percentage of unbound cytochrome *c* was calculated for three biological replicates, and statistical differences were determined via Tukey’s range test. A higher percentage of unbound cytochrome *c* represents a more positively charged population of cells.

### Protoplast generation.

Protoplast generation was as described previously with modifications (43). Cultures of OG1RF (10 ml) were grown under short-term supplementation conditions. Cultures were harvested by centrifugation (10 min, 2,739 × *g*), washed with 1× PBS, and resuspended in half (5 ml) the original volume with isotonic buffer (20% sucrose, 0.145 M NaCl, 50 mM Tris-HCl). Samples were incubated with 1 mg ml^−1^ lysozyme for 60 min at 37°C. Removal of the cell wall was verified by Gram staining.

### Anisotropy.

Protoplasts in isotonic solution were incubated with 2 nM DPH (1,6-diphenyl 1,3,5 hexatriene) at 37°C for 30 min. An Agilent Technologies Cary Eclipse fluorescence spectrophotometer was used to measure the *r* value at an excitation wavelength of 350 nm and emission wavelength of 428 nm. The observed biological range of values for anisotropy using DPH is 0.1 to 0.3, with a lower number equivalent to a more fluid membrane (25). Statistical comparisons of membrane fluidity were performed with Tukey’s range test.

### Cerulenin rescue assay.

Cultures were grown as described above for long-term supplementation with 5 μg ml^−1^ cerulenin as indicated in Fig. S3 (12). Shown are the averages and standard deviations of three biological replicates.

### Daptomycin challenge assay.

Cultures were grown and harvested using the short-term supplementation method described above, with the exception that cultures grown in the presence of serum were grown via the long-term supplementation method. Immediately after harvest, cells were spun at 2,739 × *g*, decanted, and washed with 1× PBS twice. Cells were resuspended in an equivalent volume BHI broth with 1.5 mM calcium chloride and then treated with 30 μg ml^−1^ of daptomycin (11). After addition of daptomycin, cells were incubated at 37°C; aliquots were removed at 15, 30, and 60 min and serially diluted with 0.9% sodium chloride (NaCl). The dilution series was plated on BHI agar plates and grown for 16 to 20 h at 37°C, and CFU were enumerated. The log number of survivors was plotted against time for three biological replicates, and Tukey’s range test was used to compare survival rates.

## Supplementary Material

Supplemental file 1

Supplemental file 2
