# Supplementary material for: Induction of Daptomycin Tolerance in Enterococcus faecalis by Fatty Acid Combinations
Source: Appl Environ Microbiol. 2020 Oct 1;86(20):e01178-20. doi: 10.1128/AEM.01178-20 (PMC7531955; doi:10.1128/AEM.01178-20)
Supplement: Supplemental file 1 [file AEM.01178-20-s0001.pdf]

# Induction of daptomycin tolerance in *Enterococcus faecalis* by fatty acid combinations

William Brewer, Johnathan Harrison, Holly E. Saito, and Elizabeth M. Fozo

Supplemental Data: TABLE S1, FIG S1-S4

**Table S1** Tukey's multiple comparisons test of *E. faecalis* OG1RF generation times[a]

| Condition comparison | Significance[b] | Adjusted P Value |
|---|---|---|
| **Difference in generation time with ethanol versus other conditions** | | |
| Ethanol Control vs. Oleic acid | ns | 0.2146 |
| Ethanol Control vs. Linoleic acid | ns | 0.6757 |
| Ethanol Control vs. Stearic acid | **** | <0.0001 |
| Ethanol Control vs. oleic acid and palmitic acid | ns | 0.9998 |
| Ethanol Control vs. oleic acid and stearic acid | ns | 0.9993 |
| Ethanol Control vs. linoleic acid and palmitic acid | ns | 0.9995 |
| Ethanol Control vs. linoleic acid and stearic acid | ns | 0.9900 |
| Ethanol Control vs. SLOP | ns | 0.8460 |
| Ethanol Control vs. Human Serum | ns | >0.9999 |
| **Difference in generation time with oleic acid versus other conditions** | | |
| Oleic acid vs. Linoleic acid | ns | 0.9966 |
| Oleic acid vs. Stearic acid | **** | <0.0001 |
| Oleic acid vs. oleic acid and palmitic acid | ns | 0.0692 |
| Oleic acid vs. oleic acid and stearic acid | ns | 0.0556 |
| Oleic acid vs. linoleic acid and palmitic acid | ns | 0.5584 |
| Oleic acid vs. linoleic acid and stearic acid | ns | 0.7563 |
| Oleic acid vs. SLOP | ns | 0.9707 |
| Oleic acid vs. Human Serum | ns | 0.3035 |
| **Difference in generation time with linoleic acid versus other conditions** | | |
| Linoleic acid vs. Stearic acid | **** | <0.0001 |
| Linoleic acid vs. oleic acid and palmitic acid | ns | 0.3274 |
| Linoleic acid vs. oleic acid and stearic acid | ns | 0.2781 |
| Linoleic acid vs. linoleic acid and palmitic acid | ns | 0.9602 |
| Linoleic acid vs. linoleic acid and stearic acid | ns | 0.9949 |
| Linoleic acid vs. SLOP | ns | >0.9999 |
| Linoleic acid vs. Human Serum | ns | 0.7960 |
| **Difference in generation time with stearic acid versus combinations** | | |
| Stearic acid vs. oleic acid and palmitic acid | **** | <0.0001 |
| Stearic acid vs. oleic acid and stearic acid | **** | <0.0001 |
| Stearic acid vs. linoleic acid and palmitic acid | **** | <0.0001 |
| Stearic acid vs. linoleic acid and stearic acid | **** | <0.0001 |
| Stearic acid vs. SLOP | **** | <0.0001 |
| Stearic acid vs. Human Serum | **** | <0.0001 |
| **Difference in generation time with oleic acid combinations versus other conditions** | | |
| Oleic acid and palmitic acid vs. oleic acid and stearic acid | ns | >0.9999 |
| Oleic acid and palmitic acid vs. LA & PA | ns | 0.9538 |

| | | |
|---|---|---|
| Oleic acid and palmitic acid vs. LA & SA | ns | 0.8431 |
| Oleic acid and palmitic acid vs. SLOP | ns | 0.5024 |
| Oleic acid and palmitic acid vs. Human Serum | ns | 0.9978 |
| Oleic acid and stearic acid vs. linoleic acid and palmitic acid | ns | 0.9272 |
| Oleic acid and stearic acid vs. linoleic acid and stearic acid | ns | 0.7913 |
| Oleic acid and stearic acid vs. SLOP | ns | 0.4401 |
| Oleic acid and stearic acid vs. Human Serum | ns | 0.9946 |
| **Difference in generation time with linoleic acid combinations versus other conditions** | | |
| Linoleic acid and palmitic acid vs. LA & SA | ns | >0.9999 |
| Linoleic acid and palmitic acid vs. SLOP | ns | 0.9944 |
| Linoleic acid and palmitic acid vs. Human Serum | ns | >0.9999 |
| Linoleic acid and stearic acid vs. SLOP | ns | 0.9998 |
| Linoleic acid and stearic acid vs. Human Serum | ns | 0.9983 |
| SLOP vs. Human Serum | ns | 0.9258 |

[a]Analyses were performed based on generation times presented in TABLE 1 and FIG 1 of the main text.

[b]NS: not significant based on a *P* > 0.05. **** indicates *P* < 0.0001.

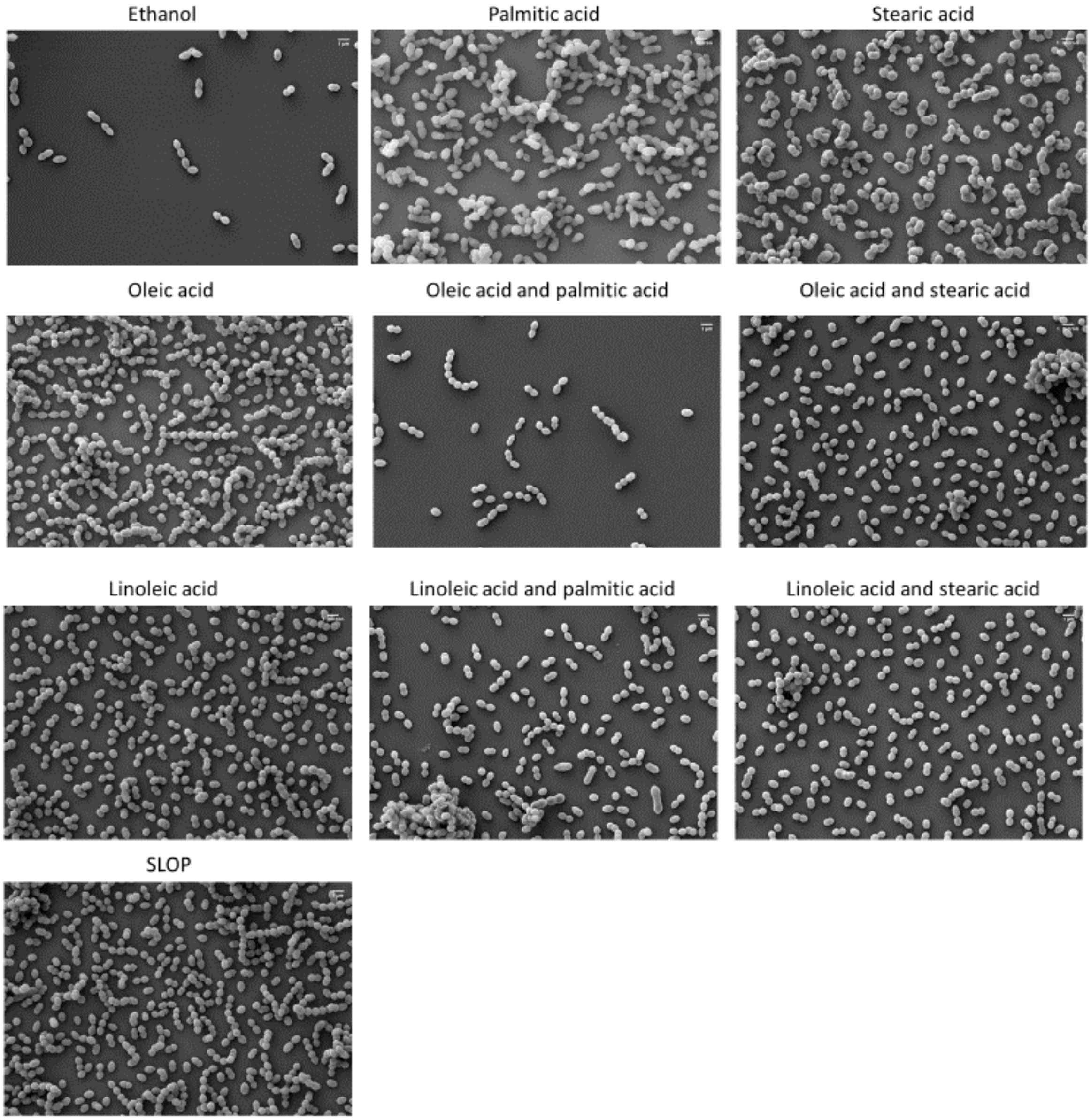


**FIG S1** SEM of OG1RF after long-term supplementation with fatty acids or solvent control (ethanol); details provided in MATERIALS AND METHODS. Images taken at 10,000 magnification at KeV 5.0. Scale bar represents 1 μm.

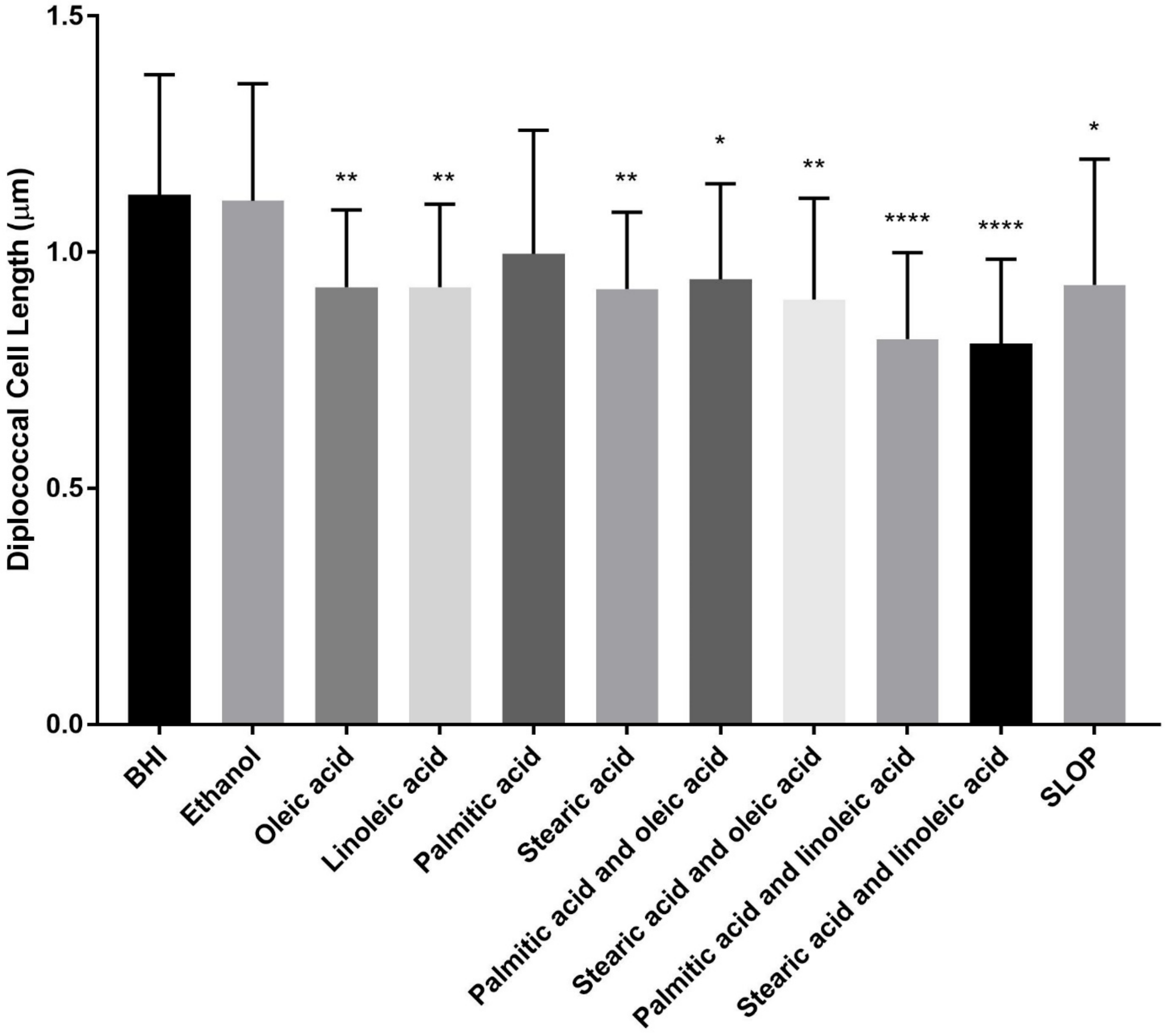


**FIG S2** Average cell length of 30 individual cells per growth condition. BHI indicates media without supplementation; all other conditions listed were added to BHI as described in the MATERIALS AND METHODS. High standard deviation is attributed to varying stages of reproduction that was captured for each sample. Asterisks indicate the following significance: * $P < 0.05$, ** $P < 0.01$, **** $P < 0.0001$ as determined via Tukey's Range Test.

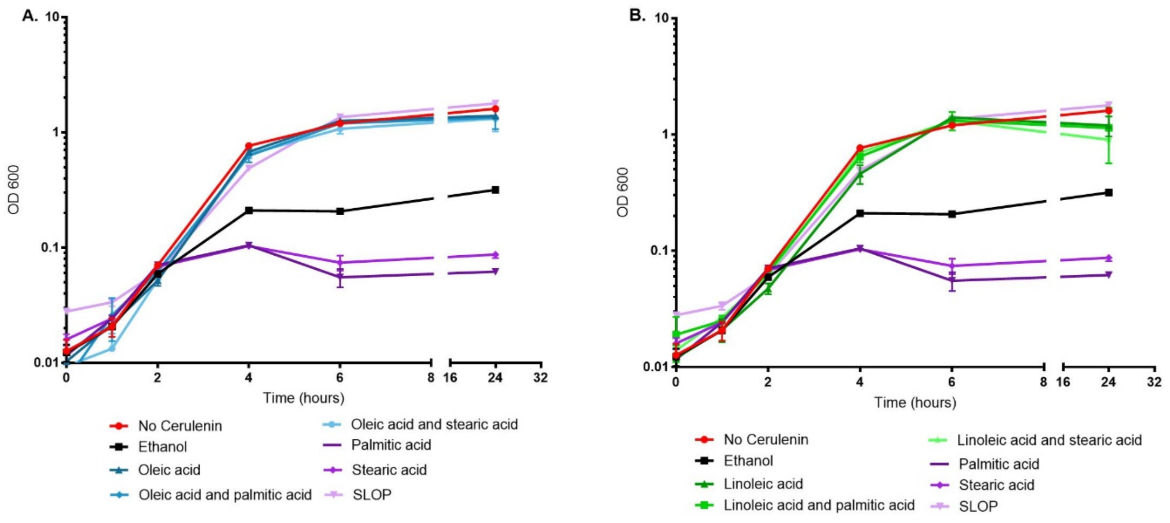


**FIG S3** Protective fatty acids rescue from inhibition of *de novo* fatty acid biosynthesis. OG1RF grown in media containing solvent control (ethanol), a fatty acid or fatty acid mixture and 5 μg $ml^{-1}$ cerulenin as indicated in MATERIAL AND METHODS. Note, the same biological replicates are re-plotted in the different panels. *n*=3 biological replicates

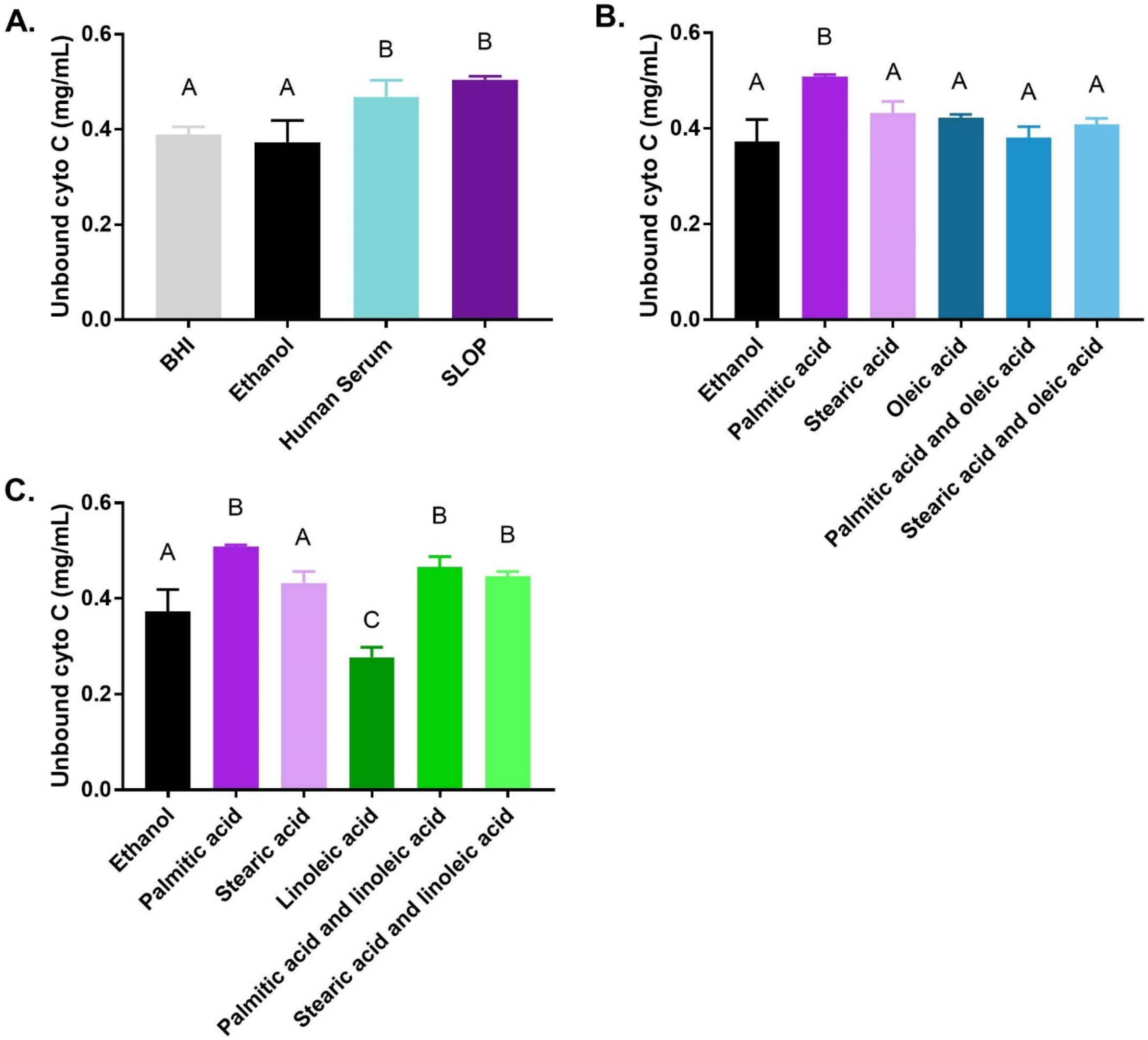


**FIG S4** Alterations in cell envelope charge upon supplementation. Interaction of positively charged cytochrome C with OG1RF: y axis indicates a more positive cell charge. For all cases, cells were supplemented with 5 μg $ml^{-1}$ of each fatty acid indicated, 15% human serum, or equivalent volume of ethanol (solvent control). BHI indicates cells grown without any supplement. (A) Human serum and SLOP; (B) oleic acid combinations; (C) linoleic acid combinations. Note the same biological replicates are re-plotted in the different panels. *n*=3 for all samples. Letter denotes groups of significance, with different letters representing a significant difference between groups, *P* <0.05, via Tukey's Range Test.
